# Montmorillonite protection of an UV-irradiated hairpin ribozyme: evolution of the RNA world in a mineral environment

**DOI:** 10.1186/1471-2148-7-S2-S2

**Published:** 2007-08-16

**Authors:** Elisa Biondi, Sergio Branciamore, Marie-Christine Maurel, Enzo Gallori

**Affiliations:** 1 Department of Animal Biology and Genetics, University of Florence, via Romana 17, 50125 – Florence, Italy; 2 Institut Jacques Monod, Laboratoire de Biochimie de l'Evolution et Adaptabilité Moléculaire, Université Paris VI, Tour 43, 2 place Jussieu, 75251 Paris Cedex 05, France

## Abstract

**Background:**

The hypothesis of an RNA-based origin of life, known as the "RNA world", is strongly affected by the hostile environmental conditions probably present in the early Earth. In particular, strong UV and X-ray radiations could have been a major obstacle to the formation and evolution of the first biomolecules. In 1951, J. D. Bernal first proposed that clay minerals could have served as the sites of accumulation and protection from degradation of the first biopolymers, providing the right physical setting for the evolution of more complex systems. Numerous subsequent experimental studies have reinforced this hypothesis.

**Results:**

The ability of the possibly widespread prebiotic, clay mineral montmorillonite to protect the catalytic RNA molecule ADHR1 (Adenine Dependent Hairpin Ribozyme 1) from UV-induced damages was experimentally checked. In particular, the self-cleavage reaction of the ribozyme was evaluated after UV-irradiation of the molecule in the absence or presence of clay particles. Results obtained showed a three-fold retention of the self-cleavage activity of the montmorillonite-protected molecule, with respect to the same reaction performed by the ribozyme irradiated in the absence of the clay.

**Conclusion:**

These results provide a suggestion with which RNA, or RNA-like molecules, could have overcame the problem of protection from UV irradiation in the RNA world era, and suggest that a clay-rich environment could have favoured not only the formation of first genetic molecules, but also their evolution towards increasingly complex molecular organization.

## Background

The hypothesis of an ancestral era during the evolution of life known as the "RNA world" [1], originally proposed by Woese [2], Orgel [3], and Crick [4], has been strengthened by the discovery of catalytic RNA molecules (ribozymes) [5,6], and by the fundamental role played by RNA in many biological processes, particularly in ribosome structure and function [7,8]. According to this hypothesis, RNA, or an RNA-like molecule, both stored genetic information and catalyzed chemical reactions, thus resolving the "chicken-and-egg" paradox of which came first, proteins or nucleic acids [9].

*In vitro *selection experiments and observations concerning natural ribozymes have demonstrated that the range of catalytic capacities of RNA is larger and more promising than previously thought [10-14]. However, in comparison with proteins, the chemical repertoire of ribozymes remains limited because of the limited chemical diversity of RNA, which is composed of only four different building blocks with few reactive functional groups. RNA chemical diversity in the RNA world could have been enhanced by the incorporation of catalytic building blocks such as imidazoles, thiols, and amino and carboxylate groups [15-17], or catalytically functional modified nucleotides [18,19]. Moreover, first RNA species could have bound exogenous molecules carrying reactive groups, such as adenine, using them as catalytic cofactors [20,21].

If the RNA world ever existed, however, it is unlikely to have developed in a dilute aqueous solution, due to the difficulty of polymerization reactions and the instability of polymers in this environment [22,13]. Moreover, with no ozone layer, the primordial Earth was likely a hostile place for newly formed macromolecules. It is now generally held that the UV radiation flux at the Earth's surface four billion years ago was approximately 100 times greater than today's, and would have been able to degrade most organic molecules [23-25]. Effects of UV irradiation on biomolecules such as DNA and RNA range from breaking of the pentose-phosphate backbone to the formation of pyrimidine dimers [26,27], and are thus considered a major obstacle to the theory of the RNA world. Several hypotheses have been proposed to solve the problem [28-31], the first being Bernal's 1951 suggestion [32]. Bernal proposed that the various steps towards the formation of very complex molecules must have required the presence of a protected confined environment, namely a clay-rich setting, where the biomolecules could originate, accumulate, and evolve while protected not only from UV irradiation, but also from other possible degrading agents.

Numerous studies have reinforced the hypothesis of a clay-mediated origin of life. These include the synthesis of nucleotide components in the presence of clay minerals [33,34] and their polymerization into oligonucleotides up to the length of a small ribozyme, with or without the need of a primer [35-37]. The work of Hanczyc *et al*. [38-40] showed that clays and other minerals can favour the spontaneous conversion of fatty acids into vesicles, with the encapsulation of clay-adsorbed RNA molecules. This suggests a simple solution to the problem of primitive compartmentalization. Clay minerals were also shown to enhance some ribozyme reactions, such as self-cleavage of hammerheads present in viroid transcripts [41].

In addition, laboratory studies on the fate of DNA in different habitats have demonstrated the role of clay minerals in its environmental protection against both biotic (i.e. nucleases) and abiotic (UV, X-ray radiation) degrading agents, still maintaining biological activities such as the ability to transform competent bacterial cells [42-44]. With regard to RNA, studies carried out in the last years have shown that clay-adsorbed RNAs are able to persist in the presence of RNases, to transmit the information contained in their sequence, and to interact with other molecules present in the environment [45,41].

The above observations suggest that clay minerals could have played a central role in the formation and preservation of ancestral genetic material on the early Earth [46,47], promoting their persistence in primordial habitats.

It is crucial to understand how nucleic acid-like molecules in clay mineral environments might have been protected and biochemically favoured to undergo specific chemical reactions, triggering the molecular evolution that led to the first cells.

With this aim, we investigated the ability of the clay mineral montmorillonite to protect a catalytic RNA molecule, the ADHR1 (Adenine Dependent Hairpin Ribozyme 1) hairpin ribozyme [21], against UV irradiation. In particular, we studied the self-cleavage reaction carried out by this ribozyme after the treatment of the molecule with UV light at 254 nm in the presence and absence of clay mineral particles.

## Results and discussion

The ability of ADHR1 to self-cleave after treatment with UV light was tested subjecting the molecules to irradiation at 254 nm in free aqueous solution, and in the presence of montmorillonite clay particles.

Two equivalent samples of ADHR1 were subjected to a denaturation/renaturation step, to obtain the most stable conformation of the molecules before treatments. Then, 17 mg/ml of montmorillonite, or the same volume in water, were added to the samples. Both preparations, with a final RNA concentration of 3 × 10^-6 ^M, were subjected to UV irradiation for 5'.

After treatment, irradiated molecules were tested for the retention of self-cleavage activity. Two parallel kinetic reactions were performed at 23°C adding a self-cleavage buffer to the samples, leading to the following final reaction conditions (as detailed in Ref. 21): 6 mM MgCl_2_, 4 mM adenine, 40 mM HEPES pH 7.5, with a final RNA concentration of 1 × 10^-6 ^M, and 5 mg/ml montmorillonite in the reaction performed on the sample treated in the presence of clay particles.

Two control kinetic reactions, in the absence and in the presence of the mineral, were also performed by subjecting ADHR1 to all the same conditions as the former ones, but without UV irradiation.

**Figure 1:**
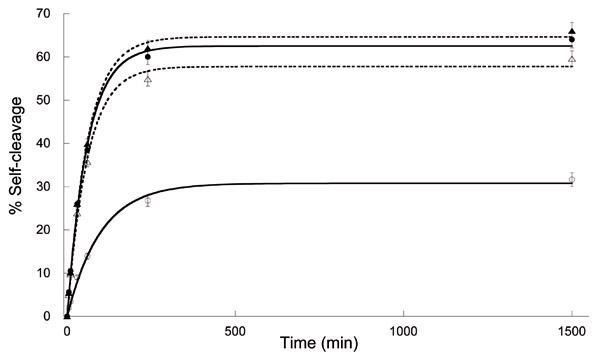
**Time course of ADHR1 self-cleavage after 5' UV irradiation in the presence and absence of montmorillonite**. Solid lines and circles represent the fittings of the two kinetics performed in free solution after no UV treatment (black circles, ●), or after irradiation (empty circles, ○). Dotted lines and triangles represent the curves of the two kinetics performed in the presence of montmorillonite after no UV treatment (black triangles, ▲), or after irradiation (△).

Samples were taken at different times: 0', 5', 10', 30', 1 h, 4 h, 24 h, quenched in equal volumes of a stop solution containing formamide and EDTA, and run on denaturing 10% PAGE. After ethidium bromide staining, the percentages of self-cleavage at each time were calculated and plotted versus time (Fig. 1). Curve fitting was performed using the equation F_t _= F_∞_(1-e−kobst
 MathType@MTEF@5@5@+=feaafiart1ev1aaatCvAUfKttLearuWrP9MDH5MBPbIqV92AaeXatLxBI9gBaebbnrfifHhDYfgasaacH8akY=wiFfYdH8Gipec8Eeeu0xXdbba9frFj0=OqFfea0dXdd9vqai=hGuQ8kuc9pgc9s8qqaq=dirpe0xb9q8qiLsFr0=vr0=vr0dc8meaabaqaciaacaGaaeqabaqabeGadaaakeaacqqGLbqzdaahaaWcbeqaaiabgkHiTiabbUgaRnaaBaaameaacqqGVbWBcqqGIbGycqqGZbWCaeqaaSGaeeiDaqhaaaaa@3638@), where F_t _is the fraction of cleaved products at time t, F_∞ _is the fraction of cleaved products at the equilibrium, and k_obs _is the observed rate constant of the reaction.

The results showed marked protection against UV irradiation performed by montmorillonite particles on ADHR1 self-cleavage. Indeed, the self-cleavage reaction of ADHR1 previously irradiated in the presence of mineral particles showed very little decrease in the reaction parameters, with 58% self-cleavage at equilibrium and k_obs _= 0.016 min^-1^, while the same parameters of its control were 64% and 0.016 min^-1^, respectively. These data correspond to less than 10% reduction of the self-cleavage at the plateau, with respect to the control reaction, and an unchanged k_obs_. In contrast, the ribozyme free in solution, compared to its control reaction, performed a slower, less-efficient catalysis, with 30% self-cleavage at equilibrium and a k_obs _= 0.010 min^-1^. The parameters for the ADHR1 control reaction free in solution (not irradiated) were roughly the same of the control in the presence of clay particles, with 63% self-cleavage at the plateau, and k_obs _= 0.017 min^-1^. Thus, a decay in both the percentage of self-cleavage at the plateau and in the observed kinetic constant was shown, calculated as a 50% and a 40% decrease, respectively.

To better analyze the effect of protection against UV light at 254 nm performed by montmorillonite on ADHR1 hairpin ribozyme, different irradiation times were also checked.

Several equal aliquots of ADHR1 were prepared as described above, with 17 mg/ml of montmorillonite, or the same volume in water, and the samples subjected to UV irradiation for the following periods: 30", 1', 3', 5', 10', 30', and 1 h.

All samples were then subjected to self-cleavage as previously described. Aliquots were taken at different times, quenched in stop solution, and loaded on denaturing 10% PAGE. Gels were stained with ethidium bromide, or SybrGold in the cases in which very few amounts of self-cleavage products were detected. Quantifications of self-cleavage products were performed as above, and data from each kinetic were fitted.

**Figure 2:**
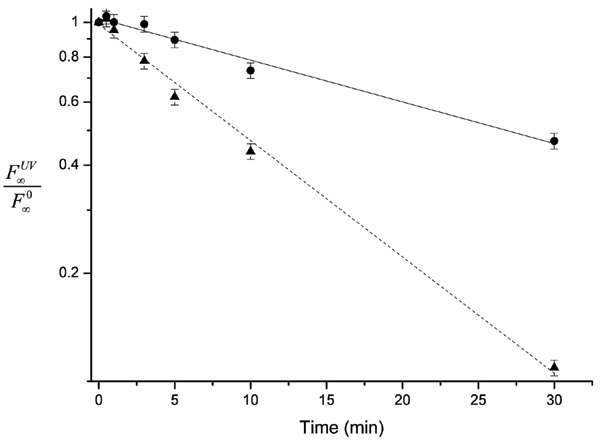
**Effects of UV irradiation at different exposure times on ADHR1 self-cleavage efficiency**. Fractions of self-cleavage products at equilibrium (F^UV^_∞_) are weighted on fractions at equilibrium calculated for the control reactions (F_∞_), and plotted versus times of UV exposure in minutes. Solid line, ● : self-cleavage data from UV-irradiated ADHR1 free in aqueous solution. Dotted line, ▲ : self-cleavage data from UV-irradiated ADHR1 in the presence of montmorillonite particles.

Fractions of cleaved products at equilibrium (F∞UV
 MathType@MTEF@5@5@+=feaafiart1ev1aaatCvAUfKttLearuWrP9MDH5MBPbIqV92AaeXatLxBI9gBaebbnrfifHhDYfgasaacH8akY=wiFfYdH8Gipec8Eeeu0xXdbba9frFj0=OqFfea0dXdd9vqai=hGuQ8kuc9pgc9s8qqaq=dirpe0xb9q8qiLsFr0=vr0=vr0dc8meaabaqaciaacaGaaeqabaqabeGadaaakeaacqqGgbGrdaqhaaWcbaGaeyOhIukabaGaeeyvauLaeeOvayfaaaaa@31C1@) calculated for each experiment, in the presence and absence of montmorillonite, were normalized by F∞0
 MathType@MTEF@5@5@+=feaafiart1ev1aaatCvAUfKttLearuWrP9MDH5MBPbIqV92AaeXatLxBI9gBaebbnrfifHhDYfgasaacH8akY=wiFfYdH8Gipec8Eeeu0xXdbba9frFj0=OqFfea0dXdd9vqai=hGuQ8kuc9pgc9s8qqaq=dirpe0xb9q8qiLsFr0=vr0=vr0dc8meaabaqaciaacaGaaeqabaqabeGadaaakeaacqqGgbGrdaqhaaWcbaGaeyOhIukabaGaeGimaadaaaaa@304B@ calculated for their own control reactions, not irradiated, with or without clay, and compared as in Fig. 2.

Results obtained clearly showed an important effect of protection of montmorillonite on the ribozyme activity. ADHR1 self-cleavage efficiency decreased exponentially with increasing times of irradiation, until only 7% self-cleavage products after 30' irradiation, and complete loss of activity with an UV treatment of 1 h. In contrast, montmorillonite-protected ADHR1 exposed to UV radiations showed far less decay of activity with increasing exposure time, retaining 30% of activity after 30' of UV exposure, and 13% after 1 h of irradiation. The time constants of the exponential decay fittings for the two sets of experiments were calculated, and gave values of 37 min and 13 min for the experiments in the presence of montmorillonite and in its absence, respectively. Thus, the life time for irradiated RNA was three times longer with montmorillonite than in free aqueous solution.

Moreover, no degradation or strand breaks of the hairpin ribozyme could be seen in any of the kinetic experiments with or without clay particles, even after long periods of exposure (data not shown). This indicates that retention of activity in the presence of montmorillonite is not due to protection from aspecific degradation, but probably to the impediment of the formation of cross-linkages in the ribozyme sequence, which could prevent the correct folding and the consequent catalysis.

**Figure 3:**
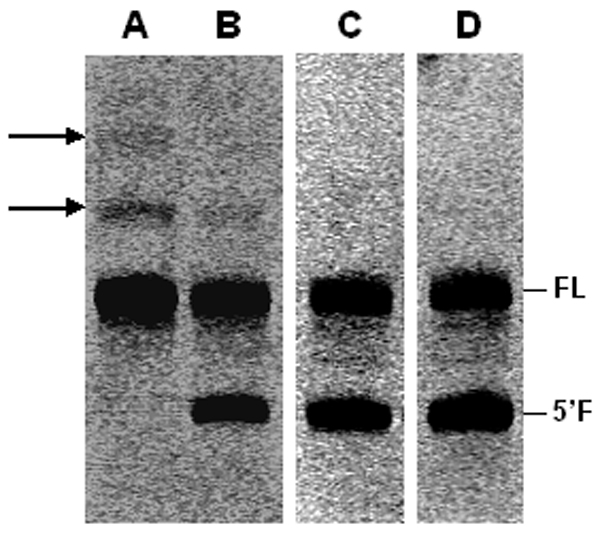
**ADHR1 24 h self-cleavage after UV irradiation in the absence or presence of montmorillonite**. (A): UV-irradiation in the absence of clay; (B): UV-irradiation in the presence of clay; (C) and (D): control 24 h self-cleavage reaction of not-irradiated ADHR1, in the absence and presence of montmorillonite, respectively. Arrows on the left indicate the two bands probably coming from UV-induced cross-linkages. FL: full-length ADHR1 molecule, 5'F: ADHR1 5' fragment of cleavage. Gel images were over-exposed to highlight the low-intensity bands.

This possibility was supported by the detection of two bands running above the one corresponding to ADHR1 in the gels of the kinetics performed after 30' and 1 h irradiation in free aqueous solution, bands which were not present (30'), or less abundant (1 h), in the corresponding reactions performed after irradiation in the presence of clay (Fig. 3). These bands could be the ADHR1 counterparts of different forms of hairpin ribozyme-substrate complexes coming from specific inter- and/or intramolecular cross-linkages which could prevent self-cleavage [48-51].

## Conclusion

In this paper, we reported the results of studies on the protection of the catalytic RNA molecule ADHR1 from UV irradiation performed by the clay mineral montmorillonite.

This aptamer, which needs adenine as a cofactor for both its self-cleavage and re-ligation reactions, is of great interest considering the prebiotic RNA world hypothesis. Indeed, adenine may itself be a prebiotic analog of histidine and could have been used by ribozymes of the RNA world in the same way as histidine is used by modern enzymes [21].

As already mentioned, in the absence of an ozone layer, the environment in which the RNA world would have originated and developed was likely exposed to a much higher UV flux. Biomolecules would have needed some form of UV shielding to protect them from this massive irradiation. These results suggest that clay minerals could have provided a mechanism for the protection of RNA, or RNA-like molecules, from UV irradiation.

Results obtained from ADHR1 self-cleavage experiments at increasing irradiation times showed significant protection against UV radiation afforded by montmorillonite particles, with a three-fold retention of the activity for the clay-protected ribozyme.

The finding of bands which could correspond to inter- and/or intra-molecular cross-linkages in the samples irradiated in free aqueous solution, but not in the ones exposed to UV-rays in the presence of montmorillonite, suggests a direct involvement of the mineral in preventing UV-induced structure rearrangements, which, in other hairpin systems, were shown to prevent correct folding and catalysis [48-51].

These studies extend and strengthen previous investigations on the biogenic properties of RNA molecules in a clay environment, aimed at assessing the possibility of the development of the RNA world on mineral substrates [45]. Such an environment could have favoured not only the formation of RNA oligomers, but also their evolution towards increasingly complex molecular organization [52,47].

It can be speculated that an RNA-like world could have arisen in microstructures present inside clay minerals, in an environment rich in phosphate and in contact with aqueous solutions containing the simple organic precursors of primordial genetic molecules. This setting could have allowed the concentration of precursors on the surface of mineral particles, the catalysis of their polymerization into macromolecules, and the protection of the resulting polymers against environmental degradation (e.g. from strong UV and X-ray radiation). This could have provided primitive genetic polymers with the time necessary to evolve towards an increasingly complex organization, triggering the molecular evolution that led to the first living cells.

## Methods

### RNA preparation

ADHR1 was *in vitro *selected in Maurel's laboratory starting from a hairpin ribozyme consensus sequence randomized in conserved regions with the method of SELEX (Systematic Evolution of Ligands by Exponential enrichment) [21]. This molecule is 85 nucleotides-long and can self-cleave, in the presence of adenine and magnesium ions, in two fragments 15 and 70 nucleotides-long, carrying the 3' and 5'-ends, respectively.

The cDNA coming from the last round of the selection procedure was PCR-amplified using *Taq *DNA-polymerase (Fermentas) with the primers P1 (5'-TAATACGACTCACTATAGGGTACGCTGAAACAG-3') and P2 (5'-CCTCCGAAACAGGACTGTCAGGGGGTACCAG-3') [21], and *in vitro *transcribed with T7 RNA-polymerase (Fermentas) over night at 37°C. The reaction mixture contained 10 ng/μl cDNA, 2 mM each rNTP, 2 mM spermidine, 10 mM DTT, 10 mM NaCl, 6 mM MgCl_2_, and 0.6 U/μl T7 RNA-polymerase. Full-length RNA transcripts were then purified by extraction from 10% denaturing polyacrilamide gel, and ethanol precipitated for three times. Purified RNA was then resuspended in double distilled water.

### Preparation of homoionic montmorillonite

The clay mineral used in the experiments was montmorillonite (M) Volclay SPV-200 (American Colloids), which is a 2:1 (Si/Al) smectite clay with a cation exchange capacity (CEC) of 76.4 cmol Kg^-1 ^and a specific surface area (SSA) of 78 m^2^g^-1^. The < 2 μm fraction of montmorillonite was made homoionic to Na^+ ^following the procedure reported by Banin *et al*. [53].

### UV irradiations

Before exposing ADHR1 molecules to UV irradiation, RNA was subjected to a denaturation/renaturation step, incubating the samples at 65°C for 3', and cooling to 23°C at the rate of 2°C min^-1^, followed by another 2' incubation at 23°C. For each sample, 50 μl of ADHR1 (4.5 × 10^-6 ^M in water) were subjected to this procedure, then 25 μl of water were added to the samples which had to be exposed to UV irradiation in the absence of the mineral, and 25 μl of montmorillonite 50 mg/ml, were added to the aliquots which had to be irradiated in the presence of clay particles. Final concentrations during irradiation were, thus, 3 × 10^-6 ^M, and 17 mg/ml for RNA and montmorillonite, respectively, in a final volume of 75 μl.

Samples prepared in this way were then transferred to 8-well glass slides, and exposed for various lengths of time to UV irradiation (Atlas Germicidal Lamp (15 W), with an intensity maximum of λ 254 nm, 4.78 × 10^-4 ^J cm^-1^) at a distance of 10 cm from the samples. Samples were mixed by pipetting every 5' to ensure homogeneity of the exposure, and 5 μl of fresh water were added every 10' to compensate for evaporation and maintain constant concentration for subsequent experiments. This quantity was evaluated by evaporation experiments (data not shown).

Samples for control reactions (not irradiated) were subjected to the same treatments, but the light used for exposure was a generic long-wavelength fluorescent lamp.

### ADHR1 self-cleavage reactions

Self-cleavage of various samples of ADHR1 (irradiated and not irradiated, with or without clay particles) was evaluated by directly adding a self-cleavage buffer to the 75 μl samples prepared as above, with the following final concentrations: 6 mM MgCl_2_, 4 mM adenine, 40 mM HEPES pH 7.5, 1 × 10^-6 ^M ADHR1, and 5 mg/ml montmorillonite in the reactions performed on the samples treated in the presence of clay particles, in a final reaction volume of 250 μl. Reactions were incubated at 23°C, 35 μl samples were taken periodically, quenched in equal volumes of a stop solution composed of 80% formamide, 30 mM EDTA pH 7.5, 0.0125% Xylene Cyanol, and directly loaded on 10%, 7 M urea denaturing polyacrylamide gels.

### Self-cleavage analysis

Band intensities from ADHR1 self-cleavage reactions were analyzed by ethidium bromide or SybrGold (Invitrogen) staining with the software ImageJ, and percentages of self-cleavage calculated as follows: % = [I_2 _/(I_1 _+ I_2_)]·100, where I_1 _and I_2 _are the intensities of the bands corresponding to the full-length molecule and its 5' fragment of cleavage, respectively. The 15 nt 3' fragment was always runned out of the gel, to better separate the other two bands.

### Statistics

Observed kinetics for ADHR1 self-cleavage were fitted with the equation F_t _= F_∞_(1 - e−kobst
 MathType@MTEF@5@5@+=feaafiart1ev1aaatCvAUfKttLearuWrP9MDH5MBPbIqV92AaeXatLxBI9gBaebbnrfifHhDYfgasaacH8akY=wiFfYdH8Gipec8Eeeu0xXdbba9frFj0=OqFfea0dXdd9vqai=hGuQ8kuc9pgc9s8qqaq=dirpe0xb9q8qiLsFr0=vr0=vr0dc8meaabaqaciaacaGaaeqabaqabeGadaaakeaacqqGLbqzdaahaaWcbeqaaiabgkHiTiabbUgaRnaaBaaameaacqqGVbWBcqqGIbGycqqGZbWCaeqaaSGaeeiDaqhaaaaa@3638@) where F_t _is the fraction of cleaved products at time t, F_∞ _is the fraction of cleaved products at the end point, and k_obs _is the observed kinetic rate constant.

Exponential decay was evaluated by fitting the data with the equation y=e−t/kd
 MathType@MTEF@5@5@+=feaafiart1ev1aaatCvAUfKttLearuWrP9MDH5MBPbIqV92AaeXatLxBI9gBaebbnrfifHhDYfgasaacH8akY=wiFfYdH8Gipec8Eeeu0xXdbba9frFj0=OqFfea0dXdd9vqai=hGuQ8kuc9pgc9s8qqaq=dirpe0xb9q8qiLsFr0=vr0=vr0dc8meaabaqaciaacaGaaeqabaqabeGadaaakeaacqqG5bqEcqGH9aqpcqWGLbqzdaahaaWcbeqaaiabgkHiTiabdsha0jabc+caViabdUgaRnaaBaaameaacqWGKbazaeqaaaaaaaa@36CC@, where *t *is the time of UV exposure, *k*_*d *_is the time constant of the exponential decay, and y represents the fraction F∞UV/F∞0
 MathType@MTEF@5@5@+=feaafiart1ev1aaatCvAUfKttLearuWrP9MDH5MBPbIqV92AaeXatLxBI9gBaebbnrfifHhDYfgasaacH8akY=wiFfYdH8Gipec8Eeeu0xXdbba9frFj0=OqFfea0dXdd9vqai=hGuQ8kuc9pgc9s8qqaq=dirpe0xb9q8qiLsFr0=vr0=vr0dc8meaabaqaciaacaGaaeqabaqabeGadaaakeaacqqGgbGrdaqhaaWcbaGaeyOhIukabaGaeeyvauLaeeOvayfaaOGaei4la8IaeeOray0aa0baaSqaaiabg6HiLcqaaiabicdaWaaaaaa@3650@, where F∞UV
 MathType@MTEF@5@5@+=feaafiart1ev1aaatCvAUfKttLearuWrP9MDH5MBPbIqV92AaeXatLxBI9gBaebbnrfifHhDYfgasaacH8akY=wiFfYdH8Gipec8Eeeu0xXdbba9frFj0=OqFfea0dXdd9vqai=hGuQ8kuc9pgc9s8qqaq=dirpe0xb9q8qiLsFr0=vr0=vr0dc8meaabaqaciaacaGaaeqabaqabeGadaaakeaacqqGgbGrdaqhaaWcbaGaeyOhIukabaGaeeyvauLaeeOvayfaaaaa@31C1@ is the fraction of cleaved product at equilibrium calculated for irradiated molecules, and F∞0
 MathType@MTEF@5@5@+=feaafiart1ev1aaatCvAUfKttLearuWrP9MDH5MBPbIqV92AaeXatLxBI9gBaebbnrfifHhDYfgasaacH8akY=wiFfYdH8Gipec8Eeeu0xXdbba9frFj0=OqFfea0dXdd9vqai=hGuQ8kuc9pgc9s8qqaq=dirpe0xb9q8qiLsFr0=vr0=vr0dc8meaabaqaciaacaGaaeqabaqabeGadaaakeaacqqGgbGrdaqhaaWcbaGaeyOhIukabaGaeGimaadaaaaa@304B@ is the fraction of cleaved product at equilibrium calculated for not-irradiated molecules.

All experiments were performed at least in triplicate. Data are expressed as mean ± standard error of the mean (S.E.M).

## Competing interests

The authors declare that they have no competing interests.

## Authors' contributions

EB designed and carried out the large part of the experimental work described on this paper. SB, M-CM, and EG equally contributed to the analysis and interpretation of the data, and to draft the manuscript. All authors read and approved the final manuscript.
